# Suspected common bile duct stones: Which test is best?

**DOI:** 10.1007/s00464-025-12054-z

**Published:** 2025-08-13

**Authors:** Andrew Varone, April Mendoza, Rondi Gelbard, Brett Tracy, Benjamin Poulose

**Affiliations:** 1 Department of Surgery, Ohio State Wexner Medical Center, 395 W 12th Ave, Columbus, OH 43210 USA; 2 Department of Surgery, UCSF-East Bay, San Francisco, CA USA; 3 Department of Surgery, University of Alabama at Birmingham, Birmingham, AL USA

**Keywords:** Choledocholithiasis, MRCP, ERCP, Common bile duct, Cholangiogram

## Abstract

**Introduction:**

The American Society for Gastrointestinal Endoscopy (ASGE) guidelines for common bile duct (CBD) stone management were created to risk stratify patients into low, intermediate, and high-risk groups to optimize interventions. Low-risk patients typically proceed to cholecystectomy with or without Intra-operative cholangiogram (IOC) whereas high-risk patients typically proceed to common duct clearance. The optimum management of intermediate-risk patients still widely varies. This study aims to characterize the use of MRCP, IOC, and endoscopic ultrasound (EUS) in patients with intermediate risk of CBD stones.

**Methods and procedures:**

This is a retrospective analysis of data from the Eastern Association for the Surgery of Trauma (EAST) Retained Common Bile Duct stone database comprised of deidentified data and outcomes of patients prospectively admitted with suspected CBD stones and/or acute biliary pancreatitis from 12 US medical centers between 2016 and 2019. Analysis of the testing characteristics sensitivity (SN), specificity (SP), and predictive values (positive (PPV) and negative (NPV)) were calculated for MRCP, EUS, and IOC based on definitive identification of CBD stones.

**Results:**

736 patients met inclusion criteria. 521 met the ASGE intermediate risk of choledocholithiasis criteria. 208/521 (39.9%) underwent pre-op MRCP, 44/521 (8.4%) underwent pre-op EUS, and 238/521 (45.7%) underwent IOC.

**Conclusions:**

There is little role for MRCP for ASGE intermediate-risk patients as this modality has both lower sensitivity and specificity than EUS and IOC. When available, either EUS or IOC is the preferred option for diagnostic clarification to identify the choledocholithiasis in this group.

Symptomatic cholelithiasis will affect approximately 10–15% of Americans in the next 5–10 years [1–4]. It is estimated that at least 10–20% of patients with symptomatic cholelithiasis will also have concurrent common bile duct stones [5, 6]. While the incidence of truly asymptomatic common bile duct stones is unknown, it is important to attempt to identify and stratify these patients to help guide their surgical management of both the gallbladder and the common bile duct. Common bile duct stones are one of the leading cause of acute pancreatitis in the US and also, when left untreated, can lead to cholangitis secondary to biliary obstruction and bacterial superinfection. These diagnoses increase patients morbidity as well as create a significant cost burden to our health care system.

Historically, patients with straightforward acute cholecystitis or biliary colic have been managed with minimally invasive cholecystectomy. Patients with clear evidence of acute cholangitis or biliary obstruction secondary to calculous disease require common bile duct drainage (either via ERCP or PTC depending on the situation), followed by cholecystectomy. There is, however, no general consensus on how to manage, or even how to identify, patients with intermediate risk of choledocholithiasis. Recognizing this, the American Society for Gastroenterology (ASGE) created a scoring system in 2010 (revised in 2019 to improve diagnostic accuracy) to help stratify these patients into low, intermediate, and high risk of choledocholithiasis and therefore help guide management [3, 7]. Low-risk patients should proceed to the operating room for cholecystectomy with or without intra-operative cholangiogram (IOC) at the surgeons discretion. High-risk patients should have planned common bile duct interrogation and clearance typically in the form of pre-operative or intra-operative ERCP. There is some evidence to support minimally invasive surgical common bile duct exploration in these ASGE high-risk patients though this still remains debated. There remains wide variability in the management of the intermediate-risk group. Some centers will plan to take most of these patients to the operating room for a cholecystectomy with a planned IOC. Others will defer definitive management to trend liver function tests. Others will obtain additional imaging to provide clarity prior to proceeding with invasive intervention. MR cholangiopancreatography (MRCP) and endoscopic ultrasound (EUS) are the two most commonly employed imaging techniques to help establish the diagnosis. Additionally, some patients will proceed to ERCP pre/intra/or post-operatively.

Endoscopic retrograde cholangiopancreatography (ERCP) has transformed the way that common bile duct obstruction is managed from a complicated, open operation to a minimally invasive, endoscopic procedure. It is now regarded as the gold standard for both diagnosis and therapy of common bile duct stones. While less morbid than open common duct exploration, it still carries a risk of serious complication including but not limited to post-ERCP pancreatitis (3–10%), bleeding (0.3–2%), and perforation (0.08–0.6%) [8]. As such, there is value to stratifying patients who potentially have common bile duct stones and reserving ERCP for those in the highest risk category.

Both EUS and MRCP have high reported diagnostic accuracies with sensitivity and specificity > 90% in the current literature [9]. EUS may slightly outperform MRCP in terms of accuracy [10] and is particularly better that MRCP at detecting smaller stones [10]. EUS is an invasive test that requires anesthesia and an experiences operator whereas MRCP is non-invasive and more widely available.

To date, no study has looked at the diagnostic accuracies of these various tests in the context of ASGE risk. Specifically, we sought to evaluate the diagnostic accuracies of these tests in the ASGE intermediate-risk patients as this is the population of patients where the current diagnostic and management paradigm remains the most unclear. This study aims to characterize the use of MRCP, IOC, and endoscopic ultrasound (EUS) in patients with intermediate risk of CBD stones and report on their diagnostic accuracy within this population to help guide future management.

## Methods

This is a retrospective analysis of data obtained in a prospective, observational study from the Eastern Association for the Surgery of Trauma (EAST) Retained Common Bile Duct Stone Study group. Patients admitted to 12 centers from 2016 to 2019 with suspected choledocholithiasis or acute biliary pancreatitis undergoing same admission cholecystectomy were included in the database. The data were deidentified of all protected health information. Patient demographics were collected including age, gender, BMI, and comorbidities. Admission vital signs and labs (complete blood count, liver function tests, and lipase) and results of transabdominal ultrasound were recorded. As part of this multi-center trial, all 12 participating institutions submitted their own data to our central database on patients admitted from 2016 to 2019 with suspected or confirmed choledocholithiasis or those undergoing cholecystectomy for acute biliary pancreatitis. Each institution received their own IRB approval and self-collected and reported data in a prospective and observational manner. Data were entered as case report forms into Research Electronic Data Capture (REDCap) which is a secure Web-based software platform that meets Health Insurance Portability and Accountability Act compliance standards. Data entries were audited and validated on an ongoing basis by the lead researcher. Any discrepancies or missing data fields were relayed back to the primary institutions for further clarifications. To our knowledge, participating centers reported all cases that met the inclusion criteria during the time frame. From these admission characteristics, patients were stratified into ASGE low-, intermediate-, and high-risk groups based on the 2019 guidelines. Patients who were ASGE intermediate risk were included in the analysis. All of the 521 intermediate patients either underwent ERCP, MRCP, IOC, or EUS for diagnosis. Patients who were ASGE low and high risk were excluded. No diagnostic or therapeutic interventions were performed to the purposes of this or any other study. Subsequent pre-op imaging findings including CT scan, MRCP, and EUS were noted as well as the subsequent patient management with pre-, intra-, or post-operative ERCP, cholecystectomy alone, cholecystectomy with IOC, or CBDE. These patients were also followed for their post-operative outcomes to identify any retained common duct stones. Results from MRCP, IOC, and EUS were compared to the gold standard of the presence of stones at the time of ERCP or CBDE. Analysis of the testing characteristics sensitivity (SN), specificity (SP), and predictive values (positive (PPV) and negative (NPV)) with 95% confidence intervals were calculated for MRCP, EUS, and IOC based on definitive identification of CBD stones.

## Results

736 patients were included in the initial dataset. 521 met the 2019 ASGE intermediate risk of choledocholithiasis criteria. 208/521 (39.9%) underwent pre-op MRCP, 44/521 (8.4%) underwent pre-op EUS, and 238/521 (45.7%) underwent IOC. 195/521(37.4%) of the intermediate-risk patients underwent pre-operative ERCP. 4 underwent intra-operative ERCP (one of whom had already underwent pre-op ERCP), and 73 underwent post-operative ERCP.

The diagnostic accuracies of MRCP, EUS, and IOC as well as their 95% CI are reported in Tables 1 and 2. MRCP had the lowest sensitivity of all three groups with 68.2%, specificity of 83.7%, PPV of 74.4%, and NPV of 79.2%.

**Table 1:** Sensitivities and specificities ASGE intermediate-risk group

Test (n)	SN	(95% CI)	SP	(95% CI)
MRCP (208)	68.2%	57.1–77.7%	83.7%	75.8–89.5%
EUS (44)	95.0%	73.1–99.7%	91.2%	71.5–98.5%
IOC (238)	78.4%	68.1–86.2%	88.7%	82.2–93.1%

**Table 2:** Positive and negative predictive values

Test (n)	PPV	(95% CI)	NPV	(95% CI)
MRCP (208)	74.4%	63.0–83.3%	79.2%	71.0–85.6%
EUS (44)	90.5%	68.2–98.3%	95.6%	76.0–99.8%
IOC (238)	80.2%	70.0–87.7%	87.5%	80.9–92.1%

## Discussion

Our study demonstrates a deviation from the previous literature in that there appears to be an inferiority of MRCP in detecting choledocholithiasis in ASGE intermediate-risk patients in comparison with EUS and IOC. This is particularly important in that the ASGE intermediate-risk population is the group of patients that MRCP is used the most to help guide their management. In our study, MRCP was used ~ 40% of the time in the ASGE intermediate-risk group. This study suggests MRCP should be used less frequently due to inferior diagnostic accuracy when compared to IOC and EUS.

Of the 208 Pre-op MRCPs, 78 of were positive. Of these, 20/78 of the patient did not have subsequent identification of stones on ERCP or IOC. Likewise, there were many instances where pre-op MRCP was negative yet the patient had positive ERCP or IOC. 130/208 of the pre-op MRCP group had a negative test; however, 27/130 ultimately had positive stones on ERCP or IOC at a later date thus leading to the low sensitivity, specificity, positive, and negative predictive values reported in the results section.

There is conflicting data in the literature regarding the optimum management from a length-of-stay (LOS) perspective in the management of patients with suspected choledocholithiasis. Lin et al. demonstrated a reduced LOS with laparoscopic cholecystectomy and IOC of 3.9 days compared to MRCP (7.0 days) and pre-ERCP (6.9 days) [11]. Ward et al. demonstrated no difference in LOS [12].

There is also a cost burden to society to consider with each of the three tests and the subsequent management of patients. Unfortunately, there is also conflicting literature reports on the optimum management strategy from this perspective depending on what factors are included in the calculation as well as the costs associated within each healthcare system. The vast majority of patients undergoing MRCP ultimately receive an invasive test (either ERCP or lap cholecystectomy) subsequently in their admission [13] which raises the question on the cost-effectiveness of this approach. Many of the modalities recommended (EUS, IOC, MRCP) are based on availability and feasibility given individual hospital resources. Use of an available modality (e.g., MRCP) may be beneficial if the alternative leads to delays in transfer for a more advantageous modality for evaluation. One reason for the inferior performance of MRCP could be due to the challenge of detecting small calculi in the distal common bile duct using this modality. Others argue that MRCP may reduce the need for unnecessary ERCP [12] thus saving cost in terms of unnecessary procedures, anesthesia, and potential complications.

Routine IOC has been proposed as the most cost-effective approach [14, 15]. Performing an IOC, however, adds additional operating time and cost to laparoscopic cholecystectomy. There may be a lost “opportunity cost” to surgeons that is hard to characterize. In addition, patients may still require post-operative ERCP as many surgeons do not routinely perform common bile duct exploration. The discussion of IOC is also important in the context of the significant increase in robotic-assisted cholecystectomy (RAC). RAC is commonly associated with fluorescent indocyanine green (ICG) cholangiography. ICG cholangiograms, while very helpful in delineating biliary anatomy, do not provide adequate examination of the common bile duct for obstruction. Traditional fluoroscopic IOC can and should be safely performed when indicated during robotic-assisted cholecystectomy. While RAC is shown to have an overall higher rate of IOC with ICG compared to laparoscopic cholecystectomy (44.2% vs 1.4%), it is associated with lower rates of traditional fluoroscopic IOC (9.1% vs 4.8%) [16]. This may have implications particularly as it pertains to our education of surgical trainees and their experience with performing IOC in their future practice.

There are some important limitations to note with this study. First, while the data were obtained prospectively, it was observational in nature and non-randomized. Patients were treated by many different providers at 12 different centers each with different resources and algorithms for managing these patients. As expected, our study demonstrated a significant amount of heterogeneity in the management of these ASGE intermediate-risk patients. This likely introduces a significant amount of bias into our data. For example, many centers have varying levels of access to ERCP which would therefore change if a surgeon was more likely to obtain a pre-op ERCP versus proceeding to the OR for a cholecystectomy with IOC. While the data in this were obtained from 12 different centers, all of them were major academic centers. Therefore, there may also be biases in patient selection, management paradigms, and resources available that would render our data not applicable or reproducible in other hospital settings. Our study did not evaluate patient comorbidities, in hospital changes in patient status, or provider decision-making which would also influence management. Additionally, while the dataset does include CBD diameter from pre-operative imaging (US, CT), there were no data regarding the size of number of stones seen on pre-op, intra-op, or post-op imaging. There were also no data collected regarding the size of CBD, size of stones, or number of stones seen on IOC or ERCP. All of this is known to play a significant role in operative decision-making.

Another important point to note is that while EUS appeared to have the best diagnostic accuracy, these numbers were very limited and therefore there was a wider confidence interval. Furthermore, EUS is invasive and a highly operator-dependent technique that requires a provider with a very advanced skill set and thus may not be realistic in all care settings.

In summary, this study does not provide a clear answer to the question regarding optimal management of the intermediate-risk patients. As mentioned above, our dataset has significant limitations which in turn limits the conclusions one can draw from our research. It does, however, shed light on the continued importance of high quality and reliable intra-operative cholangiogram as patient of the management paradigm in these patients. Furthermore, we need to ensure our surgical trainees continue to have adequate training and experience performing IOC during minimally invasive cholecystectomy.
